# Supplementary material for: Unveiling the Bioactive Potential of the Invasive Jellyfish Phyllorhiza punctata Through Integrative Transcriptomic and Proteomic Analyses
Source: Biomolecules. 2025 Aug 4;15(8):1121. doi: 10.3390/biom15081121 (PMC12383608; doi:10.3390/biom15081121)
Supplement: Supplementary file 1 [file biomolecules-15-01121-s001.zip › Suplementary Material/FileS3/PEPFOLD4/XP_065052979_2551/clusters.html]

 PEP-FOLD clusters


Download archive of all models  here   

|  |  |  |
| --- | --- | --- |
| # | file | sOPEP |
| # cluster 1 - Size: 32 |  |  |
|  | XP\_065052979\_2551-model1 | -24.4827 |
|  | XP\_065052979\_2551-model1.1 | -24.4539 |
|  | XP\_065052979\_2551-model1.2 | -24.4539 |
|  | XP\_065052979\_2551-model1.3 | -24.3908 |
|  | XP\_065052979\_2551-model1.4 | -24.3124 |
|  | XP\_065052979\_2551-model1.5 | -24.2877 |
|  | XP\_065052979\_2551-model1.6 | -24.2579 |
|  | XP\_065052979\_2551-model1.7 | -24.2097 |
|  | XP\_065052979\_2551-model1.8 | -24.2057 |
|  | XP\_065052979\_2551-model1.9 | -24.1746 |
|  | XP\_065052979\_2551-model1.10 | -24.1746 |
|  | XP\_065052979\_2551-model1.11 | -24.167 |
|  | XP\_065052979\_2551-model1.12 | -24.1482 |
|  | XP\_065052979\_2551-model1.13 | -24.1479 |
|  | XP\_065052979\_2551-model1.14 | -24.1373 |
|  | XP\_065052979\_2551-model1.15 | -24.1252 |
|  | XP\_065052979\_2551-model1.16 | -24.0998 |
|  | XP\_065052979\_2551-model1.17 | -24.0824 |
|  | XP\_065052979\_2551-model1.18 | -24.0535 |
|  | XP\_065052979\_2551-model1.19 | -24.0527 |
|  | XP\_065052979\_2551-model1.20 | -24.0159 |
|  | XP\_065052979\_2551-model1.21 | -24.0099 |
|  | XP\_065052979\_2551-model1.22 | -24.0099 |
|  | XP\_065052979\_2551-model1.23 | -23.9853 |
|  | XP\_065052979\_2551-model1.24 | -23.9831 |
|  | XP\_065052979\_2551-model1.25 | -23.9791 |
|  | XP\_065052979\_2551-model1.26 | -23.9339 |
|  | XP\_065052979\_2551-model1.27 | -23.8617 |
|  | XP\_065052979\_2551-model1.28 | -23.7878 |
|  | XP\_065052979\_2551-model1.29 | -23.6688 |
|  | XP\_065052979\_2551-model1.30 | -23.2278 |
|  | XP\_065052979\_2551-model1.31 | -21.3064 |
| # cluster 2 - Size: 3 |  |  |
|  | XP\_065052979\_2551-model2 | -19.4486 |
|  | XP\_065052979\_2551-model2.1 | -19.2382 |
|  | XP\_065052979\_2551-model2.2 | -19.1592 |
| # cluster 3 - Size: 6 |  |  |
|  | XP\_065052979\_2551-model3 | -19.2571 |
|  | XP\_065052979\_2551-model3.1 | -19.1207 |
|  | XP\_065052979\_2551-model3.2 | -18.9837 |
|  | XP\_065052979\_2551-model3.3 | -18.3271 |
|  | XP\_065052979\_2551-model3.4 | -18.2454 |
|  | XP\_065052979\_2551-model3.5 | -18.0578 |
| # cluster 4 - Size: 56 |  |  |
|  | XP\_065052979\_2551-model4 | -16.5856 |
|  | XP\_065052979\_2551-model4.1 | -16.5704 |
|  | XP\_065052979\_2551-model4.2 | -16.5604 |
|  | XP\_065052979\_2551-model4.3 | -16.5559 |
|  | XP\_065052979\_2551-model4.4 | -16.5559 |
|  | XP\_065052979\_2551-model4.5 | -16.5335 |
|  | XP\_065052979\_2551-model4.6 | -16.5266 |
|  | XP\_065052979\_2551-model4.7 | -16.5003 |
|  | XP\_065052979\_2551-model4.8 | -16.4995 |
|  | XP\_065052979\_2551-model4.9 | -16.4995 |
|  | XP\_065052979\_2551-model4.10 | -16.4881 |
|  | XP\_065052979\_2551-model4.11 | -16.4881 |
|  | XP\_065052979\_2551-model4.12 | -16.4779 |
|  | XP\_065052979\_2551-model4.13 | -16.4578 |
|  | XP\_065052979\_2551-model4.14 | -16.4566 |
|  | XP\_065052979\_2551-model4.15 | -16.4459 |
|  | XP\_065052979\_2551-model4.16 | -16.4089 |
|  | XP\_065052979\_2551-model4.17 | -16.4054 |
|  | XP\_065052979\_2551-model4.18 | -16.4051 |
|  | XP\_065052979\_2551-model4.19 | -16.4051 |
|  | XP\_065052979\_2551-model4.20 | -16.4035 |
|  | XP\_065052979\_2551-model4.21 | -16.3996 |
|  | XP\_065052979\_2551-model4.22 | -16.3736 |
|  | XP\_065052979\_2551-model4.23 | -16.3695 |
|  | XP\_065052979\_2551-model4.24 | -16.3566 |
|  | XP\_065052979\_2551-model4.25 | -16.3528 |
|  | XP\_065052979\_2551-model4.26 | -16.3395 |
|  | XP\_065052979\_2551-model4.27 | -16.339 |
|  | XP\_065052979\_2551-model4.28 | -16.3239 |
|  | XP\_065052979\_2551-model4.29 | -16.3178 |
|  | XP\_065052979\_2551-model4.30 | -16.3073 |
|  | XP\_065052979\_2551-model4.31 | -16.2998 |
|  | XP\_065052979\_2551-model4.32 | -16.2947 |
|  | XP\_065052979\_2551-model4.33 | -16.2937 |
|  | XP\_065052979\_2551-model4.34 | -16.2742 |
|  | XP\_065052979\_2551-model4.35 | -16.2727 |
|  | XP\_065052979\_2551-model4.36 | -16.2586 |
|  | XP\_065052979\_2551-model4.37 | -16.2538 |
|  | XP\_065052979\_2551-model4.38 | -16.252 |
|  | XP\_065052979\_2551-model4.39 | -16.2208 |
|  | XP\_065052979\_2551-model4.40 | -16.2206 |
|  | XP\_065052979\_2551-model4.41 | -16.2151 |
|  | XP\_065052979\_2551-model4.42 | -16.1979 |
|  | XP\_065052979\_2551-model4.43 | -16.1874 |
|  | XP\_065052979\_2551-model4.44 | -16.1852 |
|  | XP\_065052979\_2551-model4.45 | -16.185 |
|  | XP\_065052979\_2551-model4.46 | -16.182 |
|  | XP\_065052979\_2551-model4.47 | -16.177 |
|  | XP\_065052979\_2551-model4.48 | -16.1769 |
|  | XP\_065052979\_2551-model4.49 | -16.1728 |
|  | XP\_065052979\_2551-model4.50 | -16.1702 |
|  | XP\_065052979\_2551-model4.51 | -16.1582 |
|  | XP\_065052979\_2551-model4.52 | -16.1128 |
|  | XP\_065052979\_2551-model4.53 | -16.0994 |
|  | XP\_065052979\_2551-model4.54 | -16.0822 |
|  | XP\_065052979\_2551-model4.55 | -16.0513 |
| # cluster 5 - Size: 3 |  |  |
|  | XP\_065052979\_2551-model5 | -16.4454 |
|  | XP\_065052979\_2551-model5.1 | -16.2257 |
|  | XP\_065052979\_2551-model5.2 | -15.7558 |
|  |  |  |
